# Combining gene prediction methods to improve metagenomic gene annotation

**DOI:** 10.1186/1471-2105-12-20

**Published:** 2011-01-13

**Authors:** Non G Yok, Gail L Rosen

**Affiliations:** 1 Genomic Signal Processing Laboratory, Electrical and Computer Engineering, Drexel University, Philadelphia, PA 19104, USA

## Abstract

**Background:**

Traditional gene annotation methods rely on characteristics that may not be available in short reads generated from next generation technology, resulting in suboptimal performance for metagenomic (environmental) samples. Therefore, in recent years, new programs have been developed that optimize performance on short reads. In this work, we benchmark three metagenomic gene prediction programs and combine their predictions to improve metagenomic read gene annotation.

**Results:**

We not only analyze the programs' performance at different read-lengths like similar studies, but also separate different types of reads, including intra- and intergenic regions, for analysis. The main deficiencies are in the algorithms' ability to predict non-coding regions and gene edges, resulting in more false-positives and false-negatives than desired. In fact, the specificities of the algorithms are notably worse than the sensitivities. By combining the programs' predictions, we show significant improvement in specificity at minimal cost to sensitivity, resulting in 4% improvement in accuracy for 100 bp reads with ~1% improvement in accuracy for 200 bp reads and above. To correctly annotate the start and stop of the genes, we find that a consensus of all the predictors performs best for shorter read lengths while a unanimous agreement is better for longer read lengths, boosting annotation accuracy by 1-8%. We also demonstrate use of the classifier combinations on a real dataset.

**Conclusions:**

To optimize the performance for both prediction and annotation accuracies, we conclude that the consensus of all methods (or a majority vote) is the best for reads 400 bp and shorter, while using the intersection of GeneMark and Orphelia predictions is the best for reads 500 bp and longer. We demonstrate that most methods predict over 80% coding (including partially coding) reads on a real human gut sample sequenced by Illumina technology.

## Background

Analysis of environmental samples, metagenomic analysis, is defined as the characterization of microbial genomes via the direct isolation of genomic sequences from the environment without prior cultivation [1]. Environmental samples are sequenced using next-generation sequencing technologies which yield short reads lengths (100-500 base pairs) [2]. In traditional analysis, the whole genome of an organism is sequenced and assembled, then genes are predicted along this continuous sequence. In metagenomics, single genomes cannot be assembled and therefore, it is a challenge to predict genes within these short sequences [3]. Accurate gene annotation for environmental samples is needed so that genes can be classified to their correct functions, and it paves the way for functional studies in metagenomics.

Traditionally gene prediction programs can be categorized in two different groups. First, the ab initio programs, which train model parameters on known annotations in order to predict unknown annotations, are widely used in gene prediction [4]. There are a large number of ab-initio gene-finding programs, e.g.: GENIE [5], GENSCAN [6], GENEID [7], GLIMMER [8], and GeneMark [9]. The second group of gene prediction programs, known as homology-based programs, that align input sequences to the closest homologous sequence in the database to predict genes. Some popular homology-based programs are GENEWISE [10], AGenDA [11], and the well-known BLAST [12]. In addition, hybrid approaches that combine the gene prediction programs have been proposed for traditional gene annotation [13-16]. Unfortunately, it is not possible to use traditional gene prediction methods in metagenomics. Applying these conventional approaches to metagenomics is restricted by the identification of open reading frames (ORFs), which begin with a start codon and end with an in-frame stop codon [3]. Usually, genes in prokayrotes are 1000-bp in length on average [17]. Due to the short sequence length of metagenomic reads (under 500-bp from next-generation technology), they contain incomplete ORFs that lack start and/or stop codons, thus conventional ab-initio programs cannot be applied to metagenomics [3]. Similarly, homology-based approaches for gene predictions rely on databases that only contain known, and thus a limited set, of genes. Therefore, both of these categories do not work well for metagenomic fragments which are about 700 bp/less than 400 bp when produced by Sanger and next generation sequencing, respectively [18].

Therefore, recent tools have emerged to address these problems for metagenomic reads. Three programs are widely used for this purpose: Orphelia [3], MetaGene(MG) [19]/MetaGeneAnnotator(MGA) [20], and GeneMark [21]. This paper will benchmark and compare these methods. Then we demonstrate that we can boost specificity drastically by 10% by combining the programs' predictions and overall, improve accuracy by 1-4% while also improving annotation (labeling the start and stop of coding regions) by 1-8%.

## Overview of Metagenomic Gene Prediction Programs

### GeneMark (GM) [22]

Like the previous GeneMark, GeneMark for metagenomics utilizes a heuristic approach that builds a set of Markov models using a minimal amount of sequence information. The heuristic approach is used to find genes in small fragments of anonymous prokaryotic genomes and in genomes of organelles, viruses, phages and plasmids, as well as, in highly inhomogeneous genomes where adjustment of models to local DNA composition is needed. It is proven that the heuristic built model is useful for dealing with prokaryotic species whose genomic sequence information is available in small amounts. Procedures for building the heuristic models are the following:

1. Obtain the relationships between positional nucleotide frequencies and the global nucleotide frequencies as well as relationships between the amino acid frequencies and the global GC% of the training sequences.

2. Approximate the obtained relationships by the standard linear regression

3. Obtain the initial values of frequency of occurrence of each of the 61 codons by calculating the products of the three positional nucleotide frequencies of corresponding nucleotides.

4. Modify the initial value of codon frequency by the frequency of each amino acid determined by the GC content.

5. Create a codon usage table for all 61 codons.

6. Construct the 3-periodic zero order Markov model of a protein coding region using the codon usage table.

The heuristic model was built using these procedures described by using a training data that consists of 357 Bacteria and Archaea species [22]. In order to build a mixture dual model, they are further divided into two sets: (1) 38 Archaea species and 319 Bacteria species; (2) 316 Mesophilic species and 41 Thermophilic species [22].

### MGA [20]

Metagene Annotator (MGA) is an upgrade version of another software package, called MetaGene (MG) which is used in gene prediction in metagenomic sequence data. MetaGene predicts genes in two stages. First, all possible ORFs are revealed from the input sequences. Next, all ORFs are scored by their base compositions and lengths using the log-odds scoring scheme. In the log-odds scoring, the frequency of an event observed in ORFs is divided by the observed frequency in random ORFs, and a base-two logarithm of the ratio is used as the score for the event [19]. Second, an optimal (high-scored) combination of ORFs is calculated using the scores of orientations and distances of neighboring ORFs in addition to the scores for the ORFs themselves [19]. However, there are two major limitations that exist in (MG) software program: the lack of ribosomal binding site (RBS) model, and a low sensitivity to atypical genes, whose codon usages are different from those of typical genes [20]. To overcome these limitations and to improve the usability of the program, a new version of the MG called the (MGA) was developed [20]. The MGA has statistical models of prophage genes that enables it to detect lateral gene transfers or phage infections. The MGA also has an adaptable RBS model based on complementary sequences of the 30 tail of 16 S ribosomal RNA which helps it to precisely predict translation starts of genes even when input genomic sequences are short and anonymous sequences. Since the MGA is based on the algorithm of MG, it has logistic regression models of the GC content and the di-codon frequencies (di-codon models) of MG [20]. These features of the MGA remarkably improve prediction accuracies of genes on a wide range of prokaryotic genomes.

### Orphelia (Orph) [3]

Orphelia is a metagenomic ORF finding program for the prediction of protein coding genes in short fragments of DNA sequences with unknown phylogenetic origin. The Orpehelia prediction engine performs gene-finding in two stages. In the first stage, features for monocodon usage, dicodon usage and translation initiation sites are extracted from the ORF sequence using linear discriminants. In the second stage, an artificial neural network combines the sequence features with ORF length and fragment GC-content, and computes a posterior probability of an ORF to encode a protein. Its neural network is trained on randomly excised DNA fragments of a specified length from the genomes that were used for discriminant training.

## Results

To improve metagenomic gene prediction and annotation, we first analyze the three leading metagenomic gene prediction programs' sensitivity and specificity to predict whether a read contains a gene. Then, we analyze the upper-bound prediction error of the algorithms, which quantifies the error when all prediction programs mark the read incorrectly. The upper-bound error is much lower than the individual methods, demonstrating that improvements can be made if we combine the predictors. Finally, we analyze different ways of combining the prediction programs to improve prediction accuracy, sensitivity, specificity, and annotation accuracy.

### Benchmarking the three gene prediction programs

**Figure 1:**
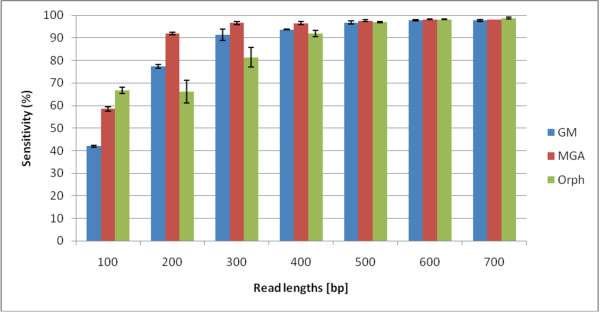
**Sensitivities of the Orphelia, MetaGeneAnnotator, and GeneMark**. The sensitivity for each of the three programs: Orphelia, MGA and GeneMark in fragment lengths 100 to 700 bp. It was generated by averaging sensitivities of 1000 random fragments from each of the four fragment types: A, B, C, and D. Orphelia has the highest sensitivity for 100 bp reads, MGA has the highest specificity for 200-400 bp reads, and all algorithms have very good sensitivity for 500 bp and longer reads.

**Figure 2:**
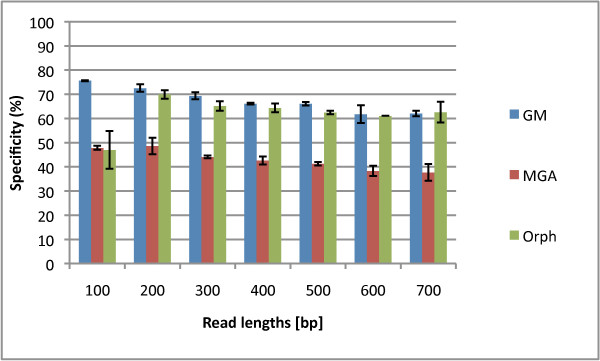
**Specificities of the Orphelia, MetaGeneAnnotator, and GeneMark**. The specificity for each of the three programs: Orphelia, MGA and GeneMark in fragment lengths 100 to 700 bp. It was generated by averaging specificities of the four fragment types: A, B, C, and D. While MGA has good sensitivity, it's specificity is generally the worst, with GeneMark generally being the best.

In this section, we aim to rigorously benchmark three different gene prediction programs for different read lengths and fragment types (fully coding/noncoding and gene edges which are defined in the Methods section). In Figures 1 and 2, we show the sensitivities and specificities of the three algorithms. MGA's sensitivities are higher than those of Orphelia and GeneMark for 200-500 bp reads. However, its specificities are the lowest, shown in Figure 2. For short reads, GeneMark does not have the highest sensitivities, but its specificities are the highest. Overall, no algorithm exceeds 80% specificity. The f-measure can indicate a combined performance of the algorithm that is not biased by the amount of training/testing data. In the supplementary material, Figure Additional file 1 we see that the GeneMark program has the best performances in terms of f-measure for most read lengths. In Figures 1 and 2, we average over the fragment types to plot sensitivity and specificity vs. read-length, but we also wish to analyze the performance for the different fragment types. In the supplementary material in Fig. Additional file 1 GeneMark's f-measure for types B and D decreases with length of the fragments, while f-measure of types A and C increases with length of the fragments. Similarly, MGA shows such a pattern too. However, Orphelia's f-measure for types A and C has similar values to types B and D.

In conclusion, we note that MGA has the best sensitivity but has the worst specificity for most read lengths. GeneMark has average sensitivity but outstanding specificity for most read lengths, which gives it a better overall f-measure for most read lengths.

**Figure 3:**
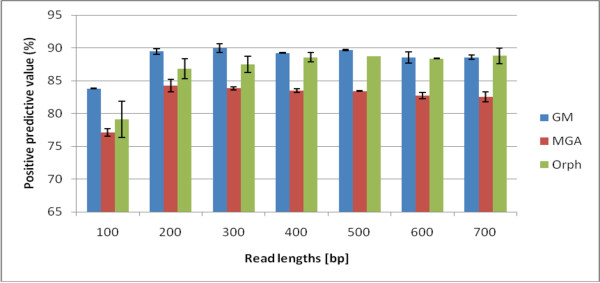
**Positive Predictive Value of of the Orphelia, MetaGeneAnnotator, and GeneMark**. The specificity computation (TP/(TP+FP) used in Hoff et al., or positive predictive value, of the three programs, Orphelia, MGA and GeneMark. This metric measures the programs' precision to correctly predict genes, as opposed to the traditional specificity value which measures a test's ability to correctly identify non-coding regions. The values here reflect similar "specificity" values found in the literature [23], [22]. The PPV metric does not fluctuate as much as the specificity because it takes into account the bias of the coding-to-non-coding ratio (75%/25%) of our simulated dataset. On the other hand, the specificity metric assesses algorithms' true ability to detect non-coding regions. In this case, GeneMark has the best PPV for short reads, while GeneMark and Orphelia are comparable for longer reads.

We note that our sensitivity and specificity measures are lower than that reported in [23], [20], and [22]. We have several reasons that this may occur. First, we have a more diverse dataset than previously studied, and we are testing with twice as many genomes as GeneMark used in their test set and eight times as many as Orphelia and MGA used. Secondly, Hoff et al. [23] uses the positive predictive value (PPV) $PPV=\frac{TP}{TP+FP}$, to benchmark their performance which measures the gene-prediction specificity of correctly predicting just the gene regions and not non-coding regions. In fact, we show that using the PPV, seen in Figure 3 and Fig. Additional file 2 instead of the traditional definition of specificity, results in high rates like previous papers. Finally, unlike GeneMark that discards hypothetical genes, we also used Genbank's hypothetical gene annotations to be as complete as possible. Also, previous methods do not describe the size of the training set (e.g. the number of reads per genome), so our sampling of ~ 40 reads per genome per read-length is quite reasonable. Also as mentioned, we take 28,000 reads of different read lengths more than any previous study has endeavored. We would like to point out to the reader that the trends are the highlight of our analysis and that the specific numbers should not be the focal point.

### Upper-bound Analysis

**Figure 4:**
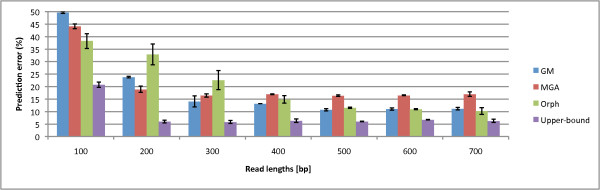
**Prediction Errors vs. Upper-bound Prediction Error**. The prediction errors of the three programs, Orphelia, MGA and GeneMark, and their upper-bound, for fragment lengths 100 to 700 bp. For 200-400 bp, Orphelia has the highest prediction errors, while MGA has the highest for longer read lengths. GeneMark has the worst prediction error for 100 bp read lengths. Also, the upper-bound analysis (which marks a read correct if at least one of the algorithms correctly predicts it) significantly reduces the error rate, showing promise for combining the methods.

Previously, we have analyzed the upper-bound prediction error of the three algorithms [24]. In this analysis, we aim to demonstrate that the three prediction programs complement each other, and we aim to show that using combinations of the three may reduce prediction error. In the upper-bound analysis for coding regions, we choose the best prediction out of the three. In other words, if all three programs incorrectly predict the read, this would contribute to the upper-bound prediction error, otherwise if one of the predictors is correct, we mark the read as correct. Through this analysis, we can also calculate the upper-bound prediction accuracy, the accuracy if at least one of the predictors was correct. In Figure 4, we see that the upper-bound prediction error is 5-25% lower than any single method. In fact, at 200 bp, the upper-bound prediction error seems to stabilize at a constant level (+/- 2% deviation). Therefore, we aim to combine predictors to significantly improve gene prediction performance.

### Combining the classifiers to improve prediction

**Figure 5:**
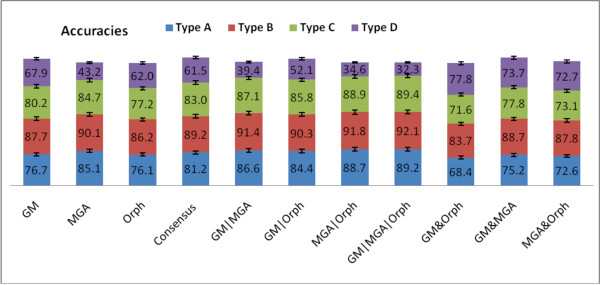
**Accuracy vs. Fragment Types for Each Method**. The accuracy for different fragment types vs. several Boolean logical combinations of the algorithms. All reads lengths for each type were averaged, resulting in an average of 7000 simulated reads for each fragment type. We can see that GM&Orph significantly improves accuracy of Type D fragments, *GM*|*MGA*|*Orph *improves the gene edges, while the consensus Boolean combination marginally improves all fragment types.

First, we aim to analyze the different types of reads, which are fully-coding, partially coding and partially non-coding, and finally non-coding. We can average over the read-lengths to see a trend in the prediction programs for each different fragment type. In Figure 5, the single-methods (GeneMark, MGA, and Orphelia) predict Type B (fully coding) fragments better than the gene edges (Type A and C). Also, the fully noncoding fragments (Type D), perform the worst. MGA performs the best on Type B. GeneMark performs the best on Type D, with Orphelia slightly behind and MGA lagging. For the combined methods, we can see that *GM&Orph *greatly improve the performance on Type D fragments, while *GM*|*MGA*|*Orph *significantly enhances prediction of fragments with gene edges (Type A and C). Lastly, the consenus method marginally enhances prediction of all fragment types.

We also investigated how the sensitivities and specificities vary for different fragment types. All programs have relatively good sensitivities for type B fragments. We compare the three programs in the supplementary material, in Figures Additional file 3 Additional file 4 and Additional file 5 and show that MGA has the highest sensitivities while Orphelia has the lowest sensitivities for types A, B and C. On the other hand, GeneMark has the best specificities among the three programs for all fragment types and read-lengths, while MGA has the lowest specificities, shown in the supplementary material in Figure Additional file 6. In order to mitigate weaknesses of these programs, we implement the Boolean logical combinations of them to combine the sensitivity vs. specificity trade-off and the Figures also show that the logical combination of GM & Orph has the best specificities. The logical combination of these classifiers shows promising results which we further investigate to find the best performance.

**Figure 6:**
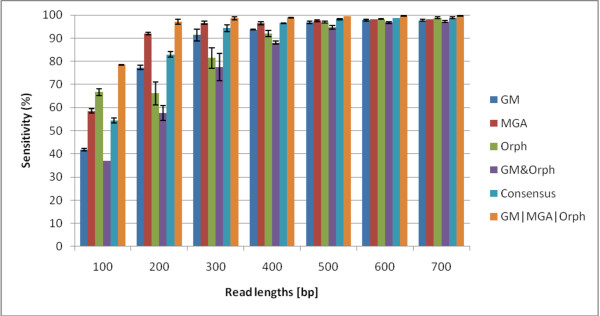
**Sensitivities for the Three Prediction Programs and Best Three Logical Combinations**. The sensitivities of the three prediction programs and the best Boolean logical combinations for read lengths 100 to 700 bp. The logical operation *GM*|*MGA*|*Orph *has the best performance and is significantly better than any single method.

**Figure 7:**
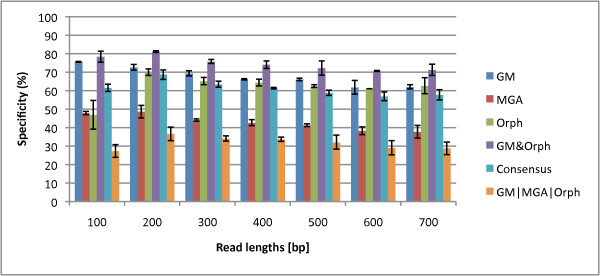
**Specificities for the Three Prediction Programs and Best Three Logical Combinations**. The specificities of the three prediction programs and the best Boolean logical combinations for read lengths 100 to 700 bp. GM & Orphelia yields significantly better performance than the closest single method, GeneMark.

**Figure 8:**
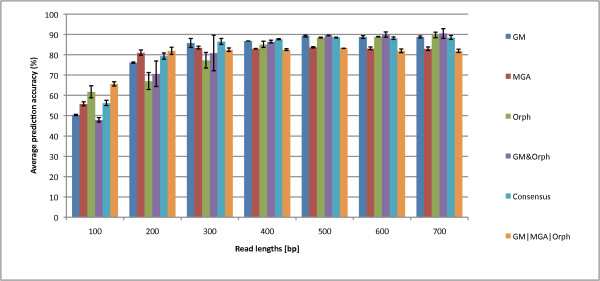
**Prediction Accuracies of the Three Gene Prediction Programs and the Best Boolean Logical Combinations**. A comparison of the prediction accuracies of the three gene prediction programs and the best Boolean logical combinations. We can see that *GM*|*MGA*|*Orph *has the best accuracy at 100 and 200 bp, the Consensus has the best accuracy at 300 and 400 bp, and GM & Orph has the best accuracy at 500 bp-700 bp. Therefore, we conclude to use different combinations depending on the read length.

We tested all the logical combinations, and we plot the best three for sensitivity, specificity, and accuracy in Figures 6, 7 and 8. Figure 6 shows that *GM*|*MGA*|*Orph *boosts sensitivity for the gene prediction. However, this combination has the lowest specificity while GM & Orph has the highest specificity at the sacrifice of lowest sensitivity. So, the question remains - what measure should we optimize for? Usually, there are more coding regions than non-coding regions, and our dataset reflects this, with 3/4 of the reads containing at least part of a gene fragment. Therefore, the accuracy measure takes this into account, and the accuracies for the combined Boolean logicals are shown in Figure 8. This plot gives us insight that different Boolean combinations have different accuracies for the various read lengths. For 100- and 200-bp reads, *GM*|*MGA*|*Orph *perform the best while the Consensus measure performs the best for 300- and 400-bp, and finally, GM & Orph performs the best for 500-700 bp. We therefore propose that the different combinations should be used for different read lengths.

We have shown that different logical combinations have better sensitivity or specificity, and this provides an advantage for some logical operations to obtain higher accuracy for longer read lengths while other combinations are better for shorter read lengths. To assess which method is best independent of read length, we varied the read length and evaluated the receiver-operating characteristic (ROC) curves for each method. We summarize the results of the ROC analysis by providing the area-under-the-curve in Table 1 (see Additional file 7 for the ROC curves). Out of the three single methods, GeneMark has the best performance. Although, by combining GM & Orphelia, this logical combination improves performance over GeneMark by 8% AUC, while providing the best accuracy on longer reads in general.

**Table 1:** AUC table for ROC analysis

Method	AUC (%)
GM	**78.2**
MGA	71.4
Orph	69.2
GM & Orph	**85.9**
Consensus	81.1
GM|MGA|Orph	65.5

The area under the curve for each method for the receiver-operatoring characteristic (ROC) curves, constructed by varying the read-length. GeneMark has the best AUC for a single-method while GM & Orphelia has the best performance for the combination methods, with the consensus combination trailing by a few percentage.

### Combining the classifiers to improve gene annotation

**Figure 9:**
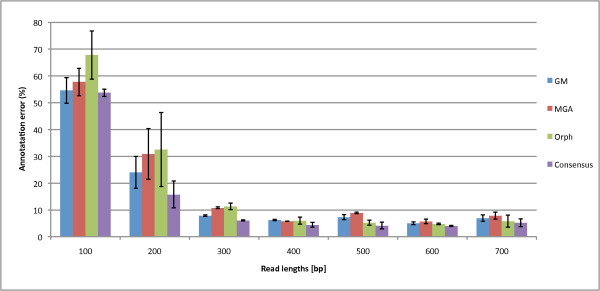
**Annotation Errors of Orphelia, MGA, GeneMark, and the Consensus combination**. The annotation errors of the three programs: Orphelia, MGA and GeneMark and the Consensus combination in reads of lengths 100 to 700 bp. Annotation refers to the program's ability to correctly label the start and stop of the gene. 1000 fragment types were used from each of the gene types A, B, and C. For the single methods, while GeneMark accuracy annotates 300 bp and shorter reads the best, Orphelia generally annotates reads 400 bp and above the best. Taking the consensus of all the methods improves the annotation error for all read lengths.

While we have previously addressed whether a read contains a gene or partial gene, we now assess the Boolean logical combinations to annotate the start and stop of the genes using the annotation error metric (inverse of annotation accuracy). If annotated inaccurately, secondary structure will most likely be incorrectly predicted, thus accurate annotation is essential. In Figure 9, we find that using the consensus of any two programs to predict gene annotation produces the lowest annotation error relative to each single program. For the single methods, GeneMark has the best annotation accuracy for short reads and Orphelia may be better for long reads. As a sidenote, we found that MGA has a tendency of predicting two or more genes on a fragment that consists of one gene.

**Figure 10:**
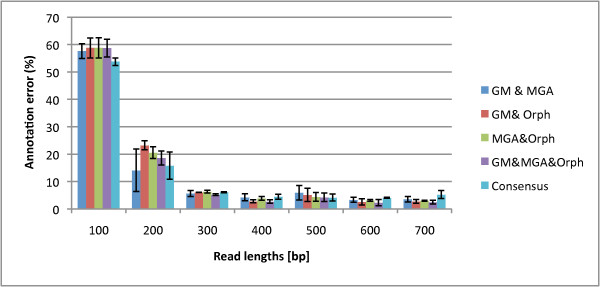
**Annotation Error of the Program Intersections**. The annotation error of the intersections for each pair of combinations and the intersections of all the methods compared to the consensus logical combination. The consensus combination has the lowest annotation error for 100 and 200 bp reads while the intersection of all three annotations (and *GM&Orph*) has the best performance for 300 bp and longer reads.

In Figure 10, the consensus combination is further compared to the logical combinations, where the best combinations for annotation error are the intersection of the annotations: MGA & GeneMark, GeneMark & Orphelia, Orphelia & MGA, and & of all three; the annotation error for the unions, or ORs, are shown in the supplementary material, Figure Additional file 8. We can see that the intersection of all three programs has the best annotation error for mostly 400 bp and longer reads. But we note that the intersection of the three programs has a trade-off between good annotation error and poor prediction accuracy (2% reduction in prediction accuracy to gain a percentage increase in annotation; see the Supplementary material in Fig. Additional file 8 and Fig. Additional file 9. While intersecting all the programs proves beneficial for the genes which the programs do predict accurately, it does not help the overall accuracy rate of intersecting the predictions. Therefore, we conclude that the consensus logical combination has the best performance for 100-400 bp since it has good prediction accuracy while maintaining good annotation error (if it lacks in predication accuracy, it is better in annotation and vice versa). GM&Orphelia is the best for 500 bp and above reads since its prediction accuracy is the best while maintaining relatively low annotation error. We provide a table for each suggested method and read length in Table 2. All data used for the prediction accuracies and annotation errors are provided in Additional file 10.

**Table 2:** Suggested method (optimizes prediction accuracy and annotation error) vs. Read-length, where annotation accuracy = 100 - annotation error

Best Method (Prediction Accuracy/Annotation Accuracy)	100 bp	200 bp	300 bp	400 bp	500 bp	600 bp	700 bp
Single Method	MGA (57%/42%)	GM (77%/76%)	GM (86%/92%)	GM (87%/93%)	Orphelia (87%/95%)	Orphelia (89%/95%)	Orphelia (90%/94%)

Combined Method	Consensus (58%/47%)	Consensus (79%/84%)	Consensus (87%/96%)	Consensus (88%/96%)	GM&Orph (89%/96%)	GM&Orph (90%/98%)	GM&Orph (91%/97%)

The best method (for combined prediction accuracy and annotation accuracy, $\frac{prediction_{-}accuracy+annotation_{-}accuracy}{2}$) vs. Read Length. The Method with Prediction Accuracy/Annotation Accuracy are in the parentheses. As shown, the Consensus gives the best overall performance for 100 bp-400 bp reads. For single methods, MGA has the best performance for 100 bp reads while GM is the best single-method for 200-400 bp length reads. For longer reads (500-700 bp), Orphelia and GM&Orphelia have the best performance.

## Discussion

### Demonstration on a real dataset, the Human Twin Lean Gut Data

**Figure 11:**
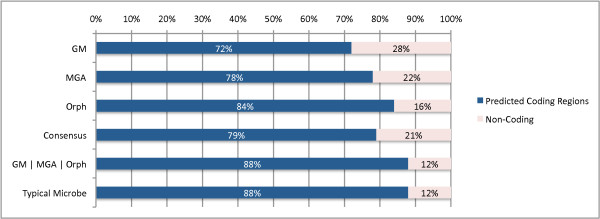
**Predicted Coding vs. Non-coding Reads in the Lean Twin Gut**. The percentage of reads of the Lean Twin Gut Sample [25] that have all/some coding sequence to the percentage of reads that are fully non-coding. Three methods result in the highest ratios: 1) the Consensus combination reveals 79%/21% coding/non-coding ratio, 2) Orphelia produces 84%/16% coding/non-coding ratio, and 3) *GM*|*MGA*|*Orphelia *classifier combination results in an 88%/12% coding/non-coding ratio.

To correctly annotate the start and stop of the genes, we previously found that a combination of all the predictors performs best for 100 bp read lengths boosting annotation accuracy by 4%. Therefore, we demonstrate the algorithm on first 20,000 Illumina reads, with average read length of 97 bp, from the distal gut from a lean human twin [25] seen in Figure 11.

For this analysis, we chose to compare against the best classifier combinations to predict coding regions in 100 bp reads - the Consensus combination and the *GM*|*MGA*|*Orph*. We can see that the *GM*|*MGA*|*Orph *combination method produces the highest gene/non-gene ratio (88% Type A/B/C and 12% for Type D). Secondly, the consensus method which was shown to have the best annotation accuracy, predicts 79% of the sequences as genes, and Orphelia falls between these two predictions with an 84% gene percentage. While the *GM*|*MGA*|*Orph *finds a similar coding/non-coding ratio found in the typical microbial genome [26,27], there is not sufficient evidence to show that a typical metagenome will represent this ratio. In the future, we plan to examine the coding/non-coding content of metagenomic samples in varying environments.

**Figure 12:**
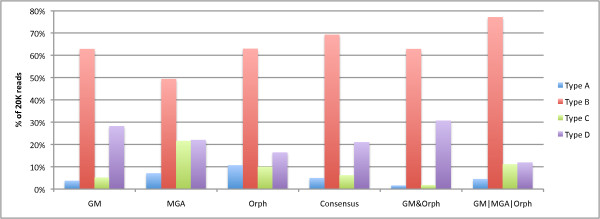
**Types for Twin Gut Microbe Population**. The percentage of reads of the sample that are of the different types described in this paper, where Type A contains the beginning of a gene, Type B contains the center of a gene, Type C contains the end of a gene, and Type D is a non-coding region. The sample was taken from the gut of a lean twin [25].

To explore the distribution of types found in the Twin Gut Microbiome population, we see Figure 12. We see that the *GM*|*MGA*|*Orph *predicts high amounts of types A, B, and C while predicting a low amount of Type D. While we cannot verify these results from real data, we believe that by combining *GM*|*MGA*|*Orph*, we can predict more of the reads as Type B instead of Type D, which results in a coding/non-coding ratio that more resembles reality. Also, we have previously shown that the consensus combination has the best annotation accuracy for the reads predicted to have coding regions, and this is reflected in Figure 12, where the amounts of Type A's and C's are almost equivalent using the consensus method (as opposed to *GM*|*MGA*|*Orph *where there are more Type C's than Type A's).

While this is beyond the scope of this paper, the next step would be to characterize and validate the extra genes discovered using the classifier combination. We propose that these coding regions may have characteristics which make them difficult to identify and may be of potential interest.

## Conclusions

We show that performances of programs, GeneMark, MetaGeneAnnotator, and Orphelia, vary for different read lengths and fragment types. The different algorithms result in a trade-off of sensitivity vs. specificity and a gradual decline in these rates for shorter reads. GeneMark's sensitivity and prediction accuracy are lower than those of Orphelia and MGA, while its average specificities are the highest for most read lengths. This is due to GeneMark's ability to correctly predict Type D fragments as non-coding. Also, GeneMark has the lowest annotation error, meaning it is the best in predicting the start and stops of genes, for short read lengths while Orphelia has the lowest annotation error for longer read lengths.

We show that we can improve on these trade-offs by combining the methods' predictions and annotations. In general, the intersection of the methods improves annotation accuracies but at the cost of poor prediction accuracies, while the union of the methods improves predictions accuracies at the cost of poor annotation. We validate the GeneMark, MGA, Orphelia, and the best combinations on a human gut sample sequenced by Illumina technology, and find that *GM*|*MGA*|*Orph *and Orphelia produce the highest coding/non-coding ratios, though more investigations are needed to determine the gene content of metagenomes. In conclusion, the consensus logical combination, or majority vote, has the best performance (optimizing prediction and annotation accuracy) for 100-400 bp while GM&Orphelia has the best performance for 500 bp and longer.

## Methods

In this work, we benchmark the performance of the three different gene prediction programs: GeneMark, MGA, and Orphelia. To do so, we simulate a dataset composed of coding, non-coding, and partially-coding metagenomic reads. Then, we describe several metrics used to compare the predictors and the parameters input into the programs. Third, we implement Boolean logical combinations of the prediction programs in order to combine their predictions to improve accuracy.

### Simulating the Metagenomic Reads

We simulated 2 samples of 28,000 artificial metagenomic fragments from 96 genomes to obtain an average and standard deviation of the figures presented in the paper. The 96 genomes, names are available in Additional file 11 consists of 19 different phyla and represents 14 Archaea species and 70 Bacterial species. If available, 7 species were randomly selected from each phyla, otherwise all the example strains were taken from the phyla. We simulated 4000 reads for each read length (100 bp, 200 bp, 300 bp, 400 bp, 500 bp, 600 bp, and 700 bp) in order to mimic a variety of sequencing technologies. For each read length, we simulated 1000 reads of each of the four different fragment types: Type A) a fragment that contains half of an upstream region and half coding region, Type B) a fragment that contains a fully coding region, Type C) a fragment that half contains the end of a coding region and contains half of a non-coding region, and Type D) a fragment that is fully a non-coding region. The coding/noncoding portions of the fragments were designed using Genbank annotations, which has been known to have errors [23], but that should not affect the overall rates. We made sure that the upstream and downstream regions in the annotations are purely non-coding regions and have no Genbank gene annotation.

**Figure 13:**
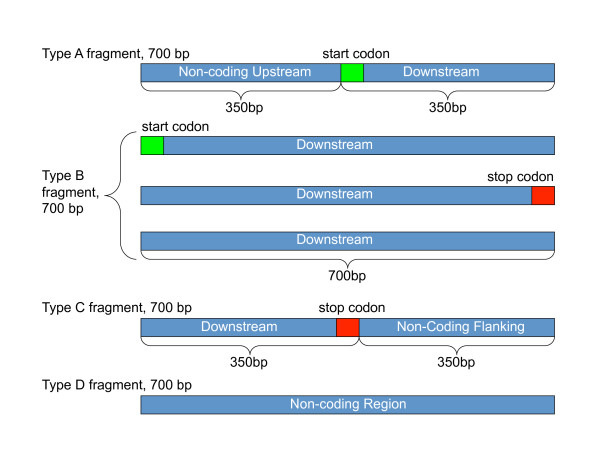
**Illustration of the four types of simulated fragments**. Illustration of the four types of simulated fragments. Type A fragments represents reads that have the first half as upstream noncoding gene regions and the latter half as downstream gene coding regions. Three different types of type B fragments are full coding regions. Type C fragments represent reads that have the first half as downstream gene coding regions and the second have as a post-gene flanking region. Type D fragments represent reads that are fully non-coding regions. The example shown here is for 700 bp reads, and the same scheme of fragmentation is used for all read lengths.

For instance, in 700 bp fragment groups, a Type A fragment consists of an upstream region of fixed length, 350 bp followed by a coding region of a fixed length 350 bp. We found 5,159 candidate fragments in our database that met this criteria. The lengths of the upstream and the coding regions are equal, but the length of the whole fragment equals to 700 bp. Type B fragments are different from type A, in that they consist purely of coding sequence, and it is picked from within a gene region. Some type B fragments may contain start or stop codons. We found a total of 145,977 candidate genes to generate Type B reads (up to 700 bp in length). Type C fragments are similar to type A fragments with the exception that the coding region comes before the flanking region in the fragment. We found a total of 5128 candidates for Type C. However, type D fragments are from non-coding regions of the DNA. We found a total of 6148 candidates for Type C. The different fragment types are illustrated in Figure 13 types of simulated fragments.

### Performance Metrics

We would like the reader to note that two types of metrics are used to assess the gene prediction programs, and this improves our study compared to previous analyses. First, the sensitivity, specificity, and f-measure is used to determine how well the algorithms predict whether or not there is a gene fragment within a read. Since sensitivity and specificity analyses require a binary detection event, we compute a true positive as detecting a gene anywhere in the read where a gene is expected, a false positive if a gene was predicted in a fully non-coding read, a false negative if a read that contained a gene is predicted as fully non-coding, and true negative as a fully non-coding read that is predicted as non-coding. Therefore, any read that contains a Type A, B, or C fragment should ideally be predicted as a gene. In our analysis, 3000 coding regions are used and 1000 noncoding regions are used. Secondly, in order to determine how well the programs annotate the start and stop of a gene, especially in Type A and C (gene edge) reads, we introduce a new measure, annotation error. The computation of annotation error compares the predicted gene coordinates to the reference gene coordinates, and annotation error is expressed as a percentage which measures how well the programs correctly predict the start/stop (correct annotation) of the gene.

We aim to analyze predictions using the sensitivity, specificity, and harmonic-mean (f-measure) measures similar to other analyses [23]. The sensitivity measure estimates the program capability of detecting reads that contain genes and is defined by the equation:

(1) $$Sensitivity=\frac{TP}{TP+FN},$$

where *TP *stands for true positives, which denotes the correct match of gene annotation between GenBank and the program. *FN *stands for false negatives, which indicates the number of overlooked genes. Next, we calculated the specificity measures from results of the three programs' gene predictions. This measure quantifies the reliability of gene prediction by the programs by assessing their ability to also identify non-coding regions [23].

(2) $$Specificity=\frac{TN}{TN+FP}$$

*FP*, stands for false positive, which is a predicted gene that does not correspond to any gene in the GenBank. And *TN *stands for true negative, which is non-coding region according to the GenBank and is confirmed to be true by the program.

By combining both measures we use the f-measure, a good indicator of overall precision and recall of the algorithms:

(3) $$F-measure=\frac{2*Sensitivity*Specificity}{Sensitivity+Specificity}$$

We compare the specificity measure to Hoff's specificity, also known as the positive predictive value.

(4) $$PPV=\frac{TP}{TP+FP}$$

While the traditional machine learning specificity measures how well an algorithm detects non-coding regions given all the non-coding regions, Hoff's specificity value tests an algorithms' ability to detect coding regions given all the predicted coding regions. The sensitivity only differs from Hoff's measure by testing an algorithms' ability to detect coding regions given all true coding regions. Therefore, the traditional specificity measure is able to now assess algorithms' abilities to detect non-coding regions. We also compute the prediction accuracy:

(5) $$Prediction\;Accuracy=\frac{TP+TN}{TP+FN+FP+TN},$$

In order to calculate the annotation error, the Genbank annotation is used as a reference. We express our gene annotation error metric:

(6) $$Annotation\;Error=\frac{\left|Lp-Lgb|+|Rp-Rgb\right|}{\left|Fgb\right|},$$

where *Lp *stands for the left end index of the gene annotation of the software program. *Lgb *stands for the left end index of the GenBank's annotation. *Rp *stands for the right end index of the fragment annotation of the program, while *Rgb *stands for the right end index of the fragment annotation of the GenBank. And |*Fgb*| stands for the fragment length according to the GenBank annotation.

### Parameters of the Programs

We benchmarked each program in May of 2010. There are required options on web submission windows for some of these programs to give gene prediction. In GeneMark's submission window we used the following setup to examine our samples: For the option of "Kingdom" given on its web page, we used "Mixture of bacteria and archaea". This option should be used for all environmental samples as well as for human and other microbiomes. The option allows for using bacterial and archaeal heuristic models concurrently. It also help in achieving high sensitivity with somewhat lower specificity. For the second option, "Model" we used "Codon Polynomial fitting order 3" for this option no temperature parameters are needed. In the output options we selected "nucleotide" and "HMM" [22].

Orphelia provides two models for scoring open reading frames in metagenomic reads, Net700 and Net300. Orphelia currently provides two models for scoring open reading frames in sequence fragments, Net700 and Net300. In order to run the web-server, the user needs to specify, which model should be used to predict genes in the input data based on the length of the input fragments. As recommended, we use Net300 for reads that are < = 300 bp and use Net700 for 400*bp *and longer reads.

MGA does not have required options to run its gene prediction engine. This can be an advantage to the novice user.

### Boolean logical combinations of the classifiers

To combine the classifiers, we form nine logical combinations of the three (where & is the AND or Intersection operation and | is the OR or UNION operation):

• *GM&MGA *(*GM*|*MGA*)

• *MGA&Orph *(*MGA*|*Orph*)

• *GM&Orph *(*GM*|*Orph*)

• *GM&MGA&Orph *(*GM*|*MGA*|*Orph*)

• *GM&MGA|MGA&Orph|GM&Orph*

The last combination we term the consensus, and it is commonly called the majority vote in the machine learning literature [28,29].

## Authors' contributions

NY developed the datasets and conducted all simulations and experiments. GR conceived the ideas behind the paper and assisted in writing. Both authors have proofread and approve of the manuscript.

## Supplementary Material

Additional file 1 **F-measures of the three gene prediction programs vs. read length**. The sensitivity for each of the three programs: GeneMark, MGA, and Orphelia in fragment lengths 100 to 700 bp. It was generated by averaging sensitivities of 1000 random fragments from each of the four fragment types: A, B, C, and D. GeneMark has the f-measure for 100 bp to 600 bp with Orphelia having the best f-measure for 700 bp. Click here for file

Additional file 2 **Positive predictive value of gene prediction programs and their best logical combinations vs. read-length**. The positive predictive values (PPV) of the 6 programs: GeneMark, MGA, Orphelia, *GM&Orph*, Consensus, and *GM*|*MGA*|*Orph *in fragment lengths 100 to 700 bp. *GM&Orph *has the best PPV. The PPV metric does not fluctuate as much as the specificity because it takes into account the bias of the coding-to-non-coding ratio (75%/25%) of our simulated dataset. On the other hand, the specificity metric assesses algorithms' true ability to detect non-coding regions. Click here for file

Additional file 3 **Comparison of the sensitivity profiles among the three programs and their logical combinations for Type A fragments**. The best performing logical combinations are chosen and are plotted against the individual programs' sensitivity profiles. The best performing logical combination varies over read lengths 100 to 700 bp. Click here for file

Additional file 4 **Comparison of the sensitivity profiles among the three programs and their logical combinations for Type B fragments**. The best performing logical combination is chosen and is plotted against the individual programs' sensitivity profiles. The best performing logical combination varies over read lengths 100 to 700 bp. Click here for file

Additional file 5 **Comparison of the sensitivity profiles among the three programs and their logical combinations for Type C fragments**. The best performing logical combination is chosen and is plotted against the individual programs' sensitivity profiles. The best performing logical combination varies over read lengths 100 to 700 bp. Click here for file

Additional file 6 **Comparison of the specificity profiles among the three programs and their logical combinations for Type D fragments**. The best performing logical combination, *GM&Orph *is chosen and is plotted against the individual programs. Click here for file

Additional file 7 **The receiver operating characteristic points for the programs GeneMark, MGA, Orphelia and their logical combinations**. The ROC is constructed by varying the read lengths. The area under the points effectively gives the average performance of the methods independent of read length. Click here for file

Additional file 8 **The annotation error vs. read length**. The annotation error is 100 *annotation accuracy*. For 100 bp and 200 bp reads, the consensus combination is the best. The consensus combination is the best compromise between prediction and annotation accuracy for short read lengths. For 400 bp-700 bp reads, the *GM&MGA&Orph *method is the best combination, with *GM&Orph *close behind. Because *GM&MGA&Orph *has very poor prediction accuracy, it is not studied in the paper, but *GM&Orph *is the best trade-off between prediction accuracy and annotation accuracy for long read lengths. Click here for file

Additional file 9 **The prediction accuracy % vs. read-length for all the methods**. OR logical combinations perform the best for 100-and 200-bp reads with the consensus combination close behind. The consensus does the best for 300 bp and 400 bp reads. *GM&Orph *performs the best for 500-700 bp. Therefore, the *GM*|*MGA*|*Orph *(a trade-off between the ORs), *GM&Orph*, and the consensus Boolean logical combinations are studied in the paper. Click here for file

Additional file 10 **Prediction Accuracy and Annotation Error table**. This le contains the tables used to construct Figure 4 and the full prediction accuracy graph and full annotation error graph found in the Appendix. Click here for file

Additional file 11 **96 organisms used for test data**. List of organisms used to simulate the metagenomic reads. Click here for file
