# Supplementary figures and images for: Combining gene prediction methods to improve metagenomic gene annotation
Source: BMC Bioinformatics. 2011 Jan 13;12:20. doi: 10.1186/1471-2105-12-20 (PMC3042383; doi:10.1186/1471-2105-12-20)

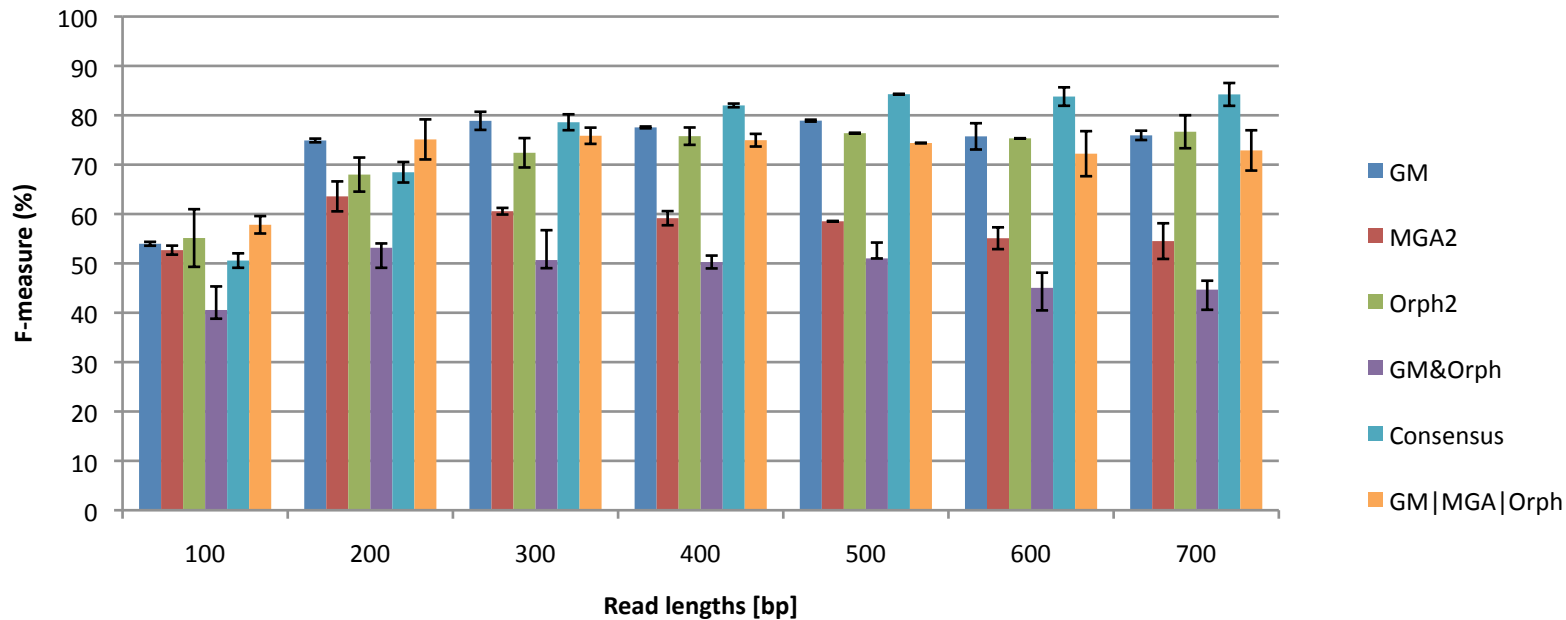

Supplement: Additional file 1 — F-measures of the three gene prediction programs vs. read length. The sensitivity for each of the three programs: GeneMark, MGA, and Orphelia in fragment lengths 100 to 700 bp. It was generated by averaging sensitivities of 1000 random fragments from each of the four fragment types: A, B, C, and D. GeneMark has the f-measure for 100 bp to 600 bp with Orphelia having the best f-measure for 700 bp. [file 1471-2105-12-20-S1.PDF]

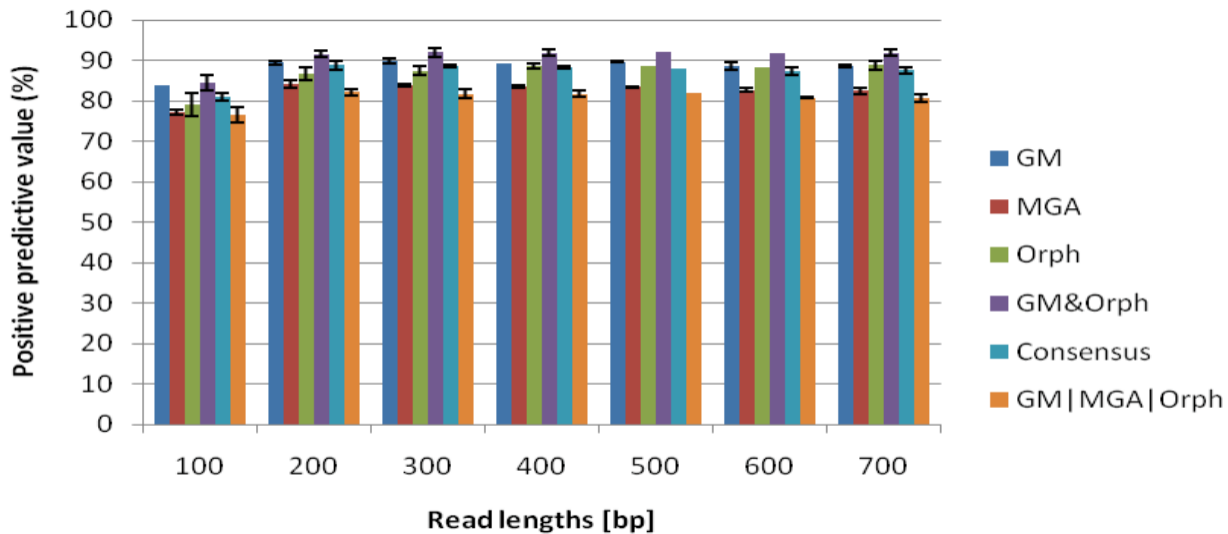

Supplement: Additional file 2 — Positive predictive value of gene prediction programs and their best logical combinations vs. read-length. The positive predictive values (PPV) of the 6 programs: GeneMark, MGA, Orphelia, GM&Orph, Consensus, and GM|MGA|Orph in fragment lengths 100 to 700 bp. GM&Orph has the best PPV. The PPV metric does not fluctuate as much as the specificity because it takes into account the bias of the coding-to-non-coding ratio (75%/25%) of our simulated dataset. On the other hand, the specificity metric assesses algorithms' true ability to detect non-coding regions. [file 1471-2105-12-20-S2.PDF]

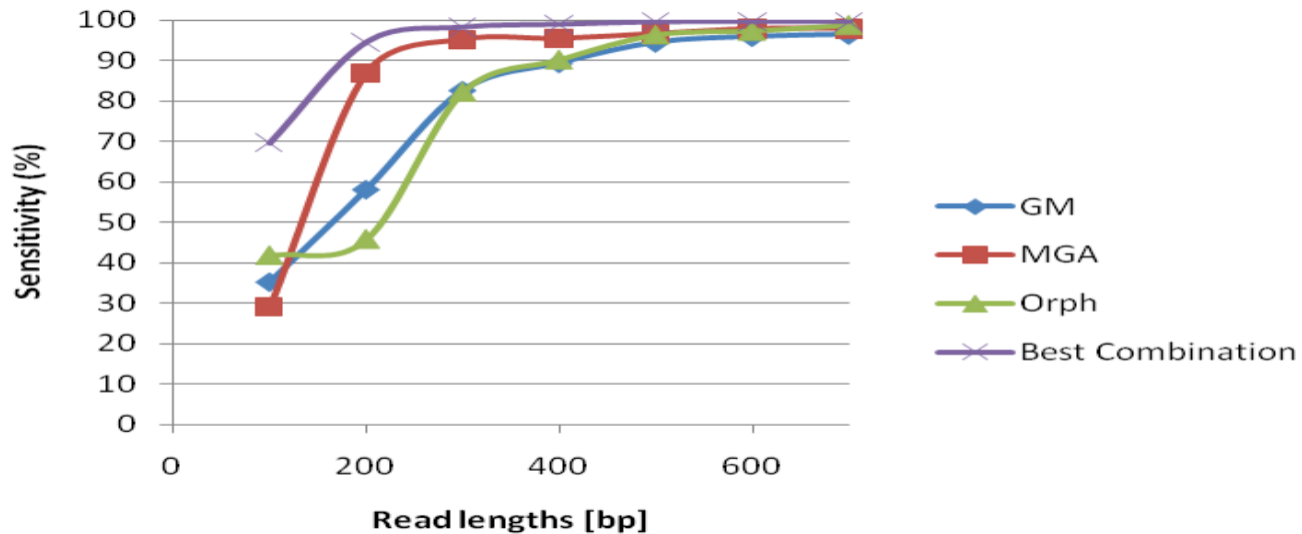

Supplement: Additional file 3 — Comparison of the sensitivity profiles among the three programs and their logical combinations for Type A fragments. The best performing logical combinations are chosen and are plotted against the individual programs' sensitivity profiles. The best performing logical combination varies over read lengths 100 to 700 bp. [file 1471-2105-12-20-S3.PDF]

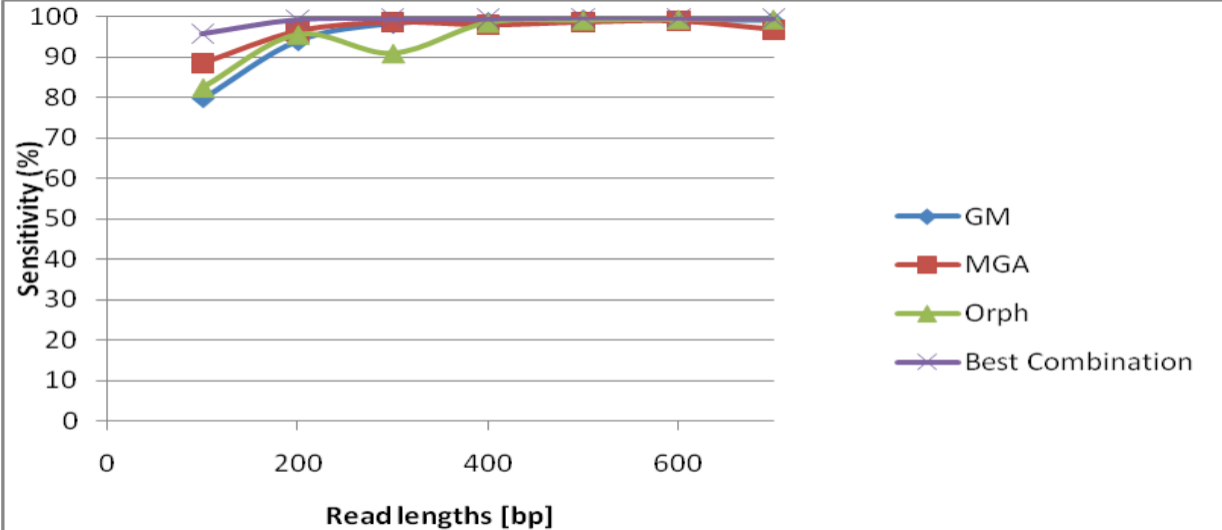

Supplement: Additional file 4 — Comparison of the sensitivity profiles among the three programs and their logical combinations for Type B fragments. The best performing logical combination is chosen and is plotted against the individual programs' sensitivity profiles. The best performing logical combination varies over read lengths 100 to 700 bp. [file 1471-2105-12-20-S4.PDF]

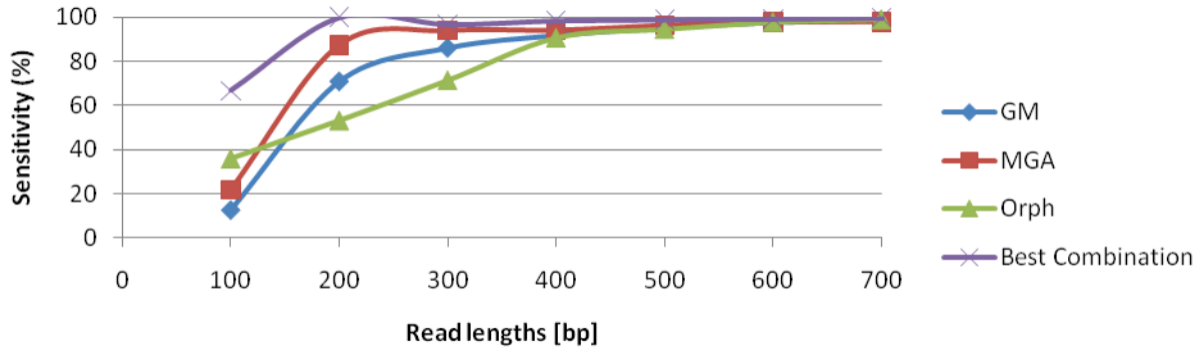

Supplement: Additional file 5 — Comparison of the sensitivity profiles among the three programs and their logical combinations for Type C fragments. The best performing logical combination is chosen and is plotted against the individual programs' sensitivity profiles. The best performing logical combination varies over read lengths 100 to 700 bp. [file 1471-2105-12-20-S5.PDF]

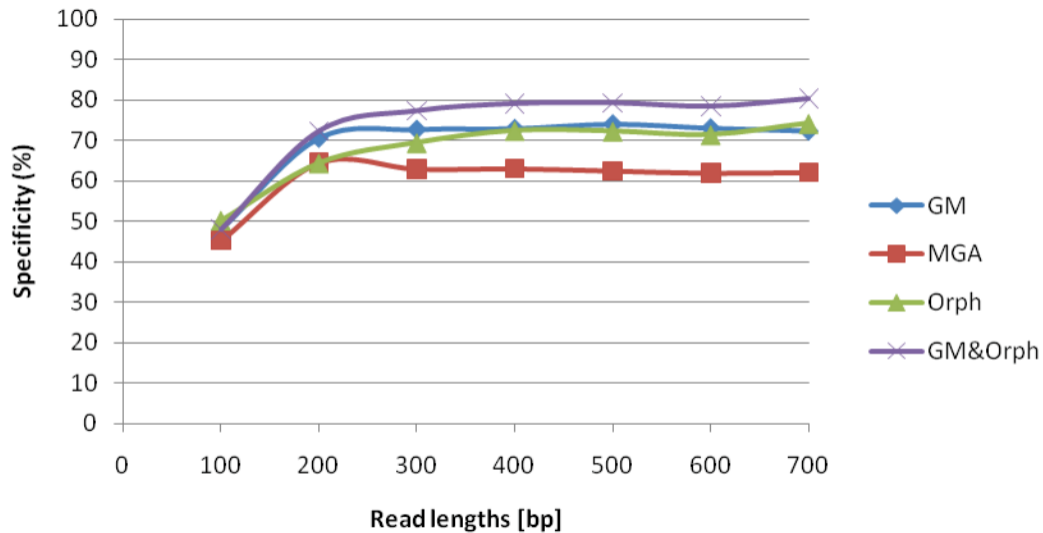

Supplement: Additional file 6 — Comparison of the specificity profiles among the three programs and their logical combinations for Type D fragments. The best performing logical combination, GM&Orph is chosen and is plotted against the individual programs. [file 1471-2105-12-20-S6.PDF]

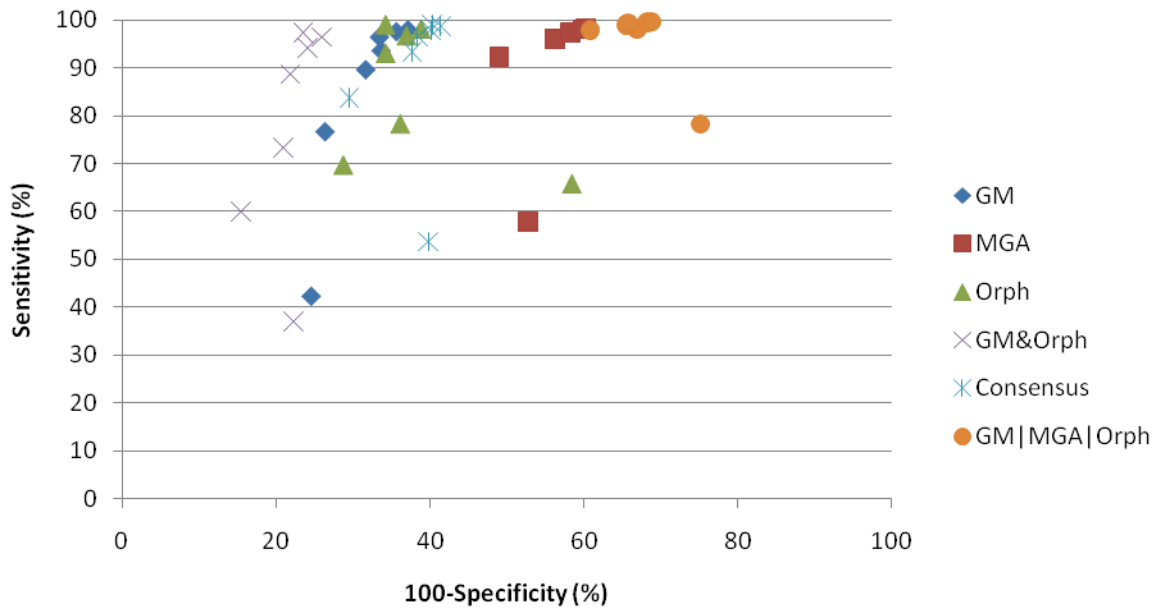

Supplement: Additional file 7 — The receiver operating characteristic points for the programs GeneMark, MGA, Orphelia and their logical combinations. The ROC is constructed by varying the read lengths. The area under the points effectively gives the average performance of the methods independent of read length. [file 1471-2105-12-20-S7.PDF]

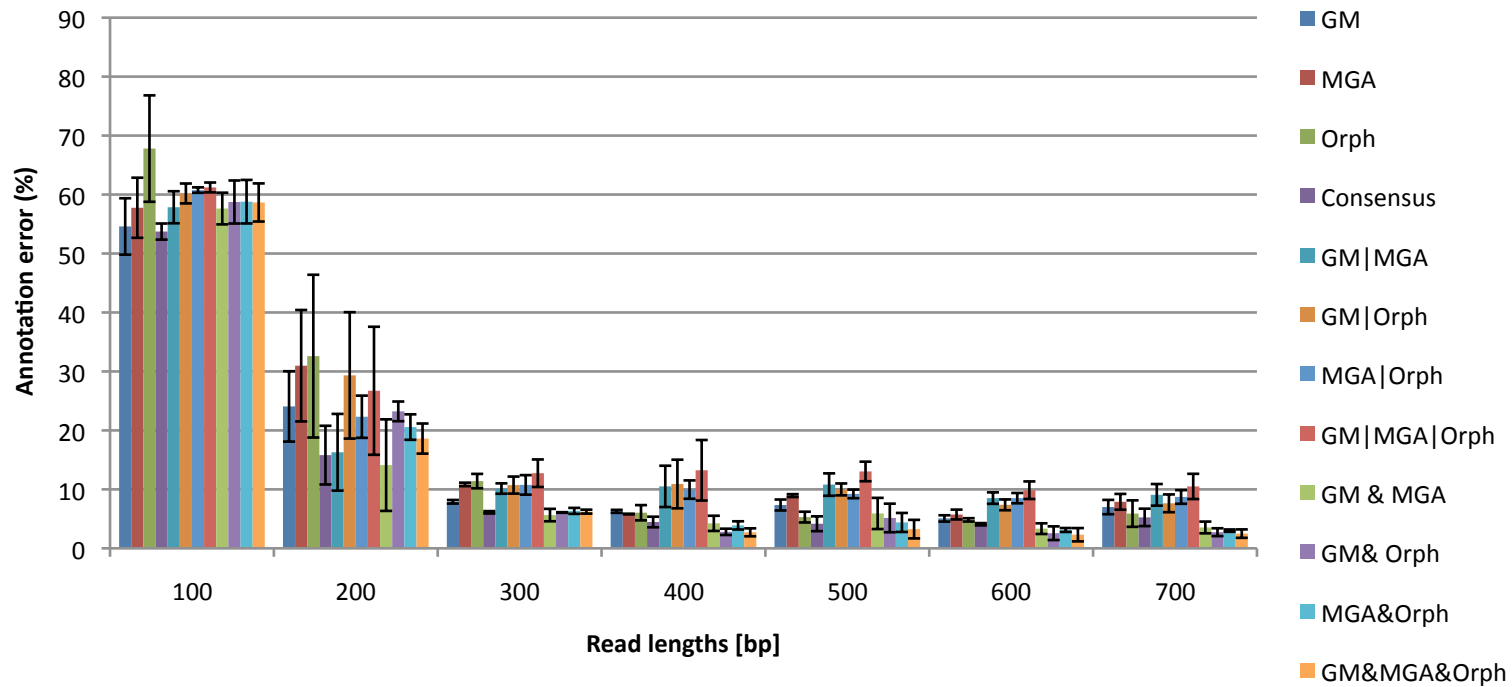

Supplement: Additional file 8 — The annotation error vs. read length. The annotation error is 100 annotation accuracy. For 100 bp and 200 bp reads, the consensus combination is the best. The consensus combination is the best compromise between prediction and annotation accuracy for short read lengths. For 400 bp-700 bp reads, the GM&MGA&Orph method is the best combination, with GM&Orph close behind. Because GM&MGA&Orph has very poor prediction accuracy, it is not studied in the paper, but GM&Orph is the best trade-off between prediction accuracy and annotation accuracy for long read lengths. [file 1471-2105-12-20-S8.PDF]

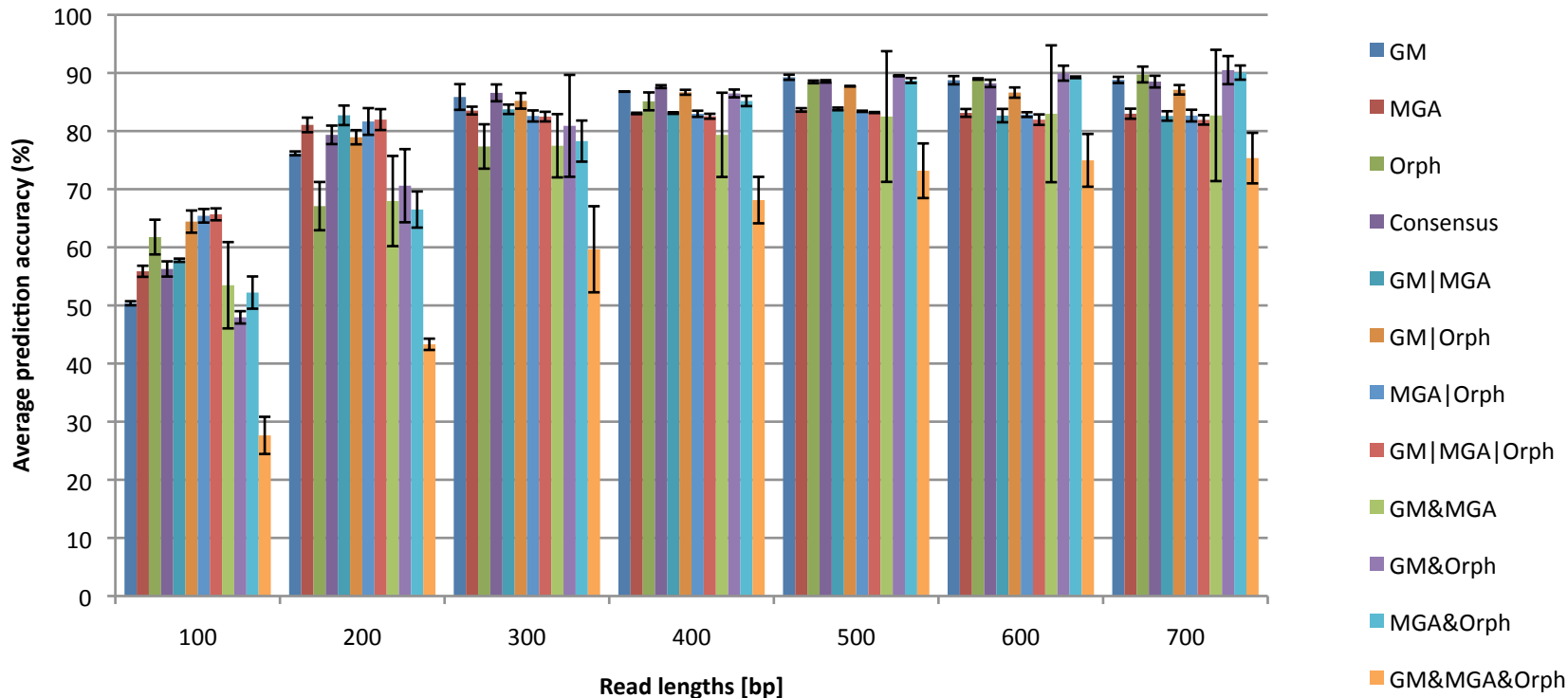

Supplement: Additional file 9 — The prediction accuracy % vs. read-length for all the methods. OR logical combinations perform the best for 100-and 200-bp reads with the consensus combination close behind. The consensus does the best for 300 bp and 400 bp reads. GM&Orph performs the best for 500-700 bp. Therefore, the GM|MGA|Orph (a trade-off between the ORs), GM&Orph, and the consensus Boolean logical combinations are studied in the paper. [file 1471-2105-12-20-S9.PDF]
